# Falls and fracture risk screening in primary care: update and validation of a postal screening tool for community dwelling older adults recruited to UK Prevention of Falls Injury Trial (PreFIT)

**DOI:** 10.1186/s12877-022-03649-5

**Published:** 2023-01-23

**Authors:** Julie Bruce, Anower Hossain, Chen Ji, Ranjit Lall, Susanne Arnold, Emma Padfield, Martin Underwood, Sarah E. Lamb

**Affiliations:** 1 Warwick Clinical Trials Unit, Warwick Medical School, University of Warwick, Coventry, CV4 7AL UK; 2 University Hospitals Coventry and Warwickshire, Coventry, CV2 2DX UK; 3 South Cloisters, University of Exeter, St Luke’s Campus, Heavitree Road, Exeter, EX1 2LU UK

**Keywords:** Falls prediction, Risk prediction, Screening, Prognostic model development, External validation

## Abstract

**Background:**

Postal screening has not previously been validated as a method for identifying fall and fracture risk in community-dwelling populations. We examined prognostic performance of a postal risk screener used in the UK Prevention of Falls Injury Trial (PreFIT; ISRCTN71002650), to predict any fall, recurrent falls, and fractures over 12 months. We tested whether adding variables would improve screener performance.

**Methods:**

Nine thousand eight hundred and eight community-dwelling participants, aged 70 years and older, and 63 general practices in the UK National Health Service (NHS) were included in a large, pragmatic cluster randomised trial comparing screen and treat fall prevention interventions. The short postal screener was sent to all participants in the trial intervention arms as an A4 sheet to be completed and returned to the GP (*n* = 6,580). The postal screener items were embedded in the baseline pre-randomisation postal questionnaire for all arms of the trial (*n* = 9,808). We assessed discrimination and calibration using area under the curve (AUC). We identified additional predictors using data from the control arm and applied these coefficients to internal validation models in the intervention arm participants. We used logistic regression to identify additional predictor variables.

**Findings:**

A total of 10,743 falls and 307 fractures were reported over 12 months. Over one third of participants 3,349/8,136 (41%) fell at least once over 12 month follow up. Response to the postal screener was high (5,779/6,580; 88%). Prediction models showed similar discriminatory ability in both control and intervention arms, with discrimination values for any fall AUC 0.67 (95% CI 0.65 to 0.68), and recurrent falls (AUC 0.71; 95% CI 0.69, 0.72) but poorer discrimination for fractures (AUC 0.60; 95% CI 0.56, 0.64). Additional predictor variables improved prediction of falls but had modest effect on fracture, where AUC rose to 0.71 (95% CI 0.67 to 0.74). Calibration slopes were very close to 1.

**Conclusion:**

A short fall risk postal screener was acceptable for use in primary care but fall prediction was limited, although consistent with other tools. Fracture and fall prediction were only partially reliant on fall risk although were improved with the additional variables.

**Supplementary Information:**

The online version contains supplementary material available at 10.1186/s12877-022-03649-5.

## Background

Falls are a major public health problem leading to injury, disability, and death. The annual incidence of multiple or recurrent fallers in community-dwelling older adults ranges from 11% to 15% [1]. Falls contribute to fracture injury and are associated with increased healthcare costs [2]. Although UK and international guidance [3–6] recommend opportunistic screening of older people when they present to healthcare services, there is lack of consensus regarding which fall screening tool to use. Numerous tools have been published, designed for use in different clinical settings (acute inpatient care, emergency departments, rehabilitation and residential care) or with specific patient populations e.g. Parkinson’s disease, stroke [7, 8].

Age alone is not a sufficient criterion for risk. Falls history, objective testing of strength, balance and reaction time, assessment of polypharmacy and vision testing have been incorporated into screening tools. The Falls Risk Assessment Tool [9], the Performance-Orientated Mobility Assessment [10], the Timed Up and Go Test [11], the Falls Risk for Older People in the Community [12] are widely used in community settings but all require face to face observation and assessment. Similarly, the Falls Risk Assessment & Screening Tool (FRAST) was developed as being suitable for administration in primary care by minimally trained staff, with onward action to be initiated by a trained professional for those identified at risk [13]. The FRAST was developed after a comprehensive review of 300 articles, from which 15 risk factors were selected. Despite often being described as simple, many face-to-face tools are quite detailed, incorporating calculations from other scales to generate overall scores e.g. FRAST incorporates scores from the Modified Falls Efficacy Scale and Timed Up and Go Test. Few falls screeners consider fracture risk as this generally involves more comprehensive assessment of individual and clinical risk factors [14, 15], some of which require bone mineral density measurement.

Impairments of activities of daily living (ADLs) have been associated with risk of falling but are rarely incorporated into screening tools. An American cohort study found that ADLs were predictive of both single and recurrent falls [16]. Similarly, Brown et al. [17] found a four-fold increased risk of falling in those with severe instrumental ADL limitation compared to those with no ADL limitations.

More detailed approaches are costly and time-consuming for delivery in primary care and are impractical for screening at a population level. We used a short, postal screener to identify falls risk in a large cohort of older people recruited to a clinical trial, the UK Prevention of Falls Injury Trial (PreFIT; ISRCTN71002650) [18]. The PreFIT screener was used to classify trial participants into lower or higher risk of falling for the purposes of referral into active trial interventions (exercise or multifactorial assessment) [19, 20]. The original study informing development of the PreFIT postal screener was the Women’s Health and Aging Study (WHAS) cohort study, conducted in Baltimore, USA [21]. Although risk items were derived and internally validated in the WHAS cohort, the screener had yet to be tested in an English population and administered in postal format. In this post-hoc analyses, we examined postal screener performance in both the control and interventions arms in PreFIT. We presented unadjusted results for the screener performance in our main report of the PreFIT trial. Here we expand and detail our approach to screening tool development in line with current best practice, and present final adjusted models. No differences were found between rates of falls or fractures at 12 months by treatment arms in PreFIT [19], hence trial data were restructured for these analyses.

### Aim of study

To estimate uptake and performance of a short postal risk screener used in a UK sample of older adults to predict future falls and fractures over 12 months, and to determine whether additional variables could improve prediction.

## Methods

### Overview

We adhered to the Transparent Reporting of a multivariate prediction model for Individual Prognosis or Diagnosis (TRIPOD) statement [18]. In the trial control arm, we used screener items that were embedded in the postal baseline questionnaire to allow us to make estimates of predictive performance without any possibility of confounding by treatment response (Model 1). We estimated screener performance in the combined intervention arms, which allowed direct assessment of the performance in the exact mode of administration intended for clinical practice, but with the possibility of event rates being influenced by treatment and response to the screener (Model 2). We also investigated whether sociodemographic and health-related variables might further improve prognostic prediction of any fall or fracture over 12 months for future studies (Models 3 and 4).

### Source data for postal screener development

The postal screener algorithm was developed in the USA WHAS cohort that included an age-stratified random sample of 1,002 women aged ≥ 65 years, representing the one third most disabled women living in the community [22]. A wide range of candidate variables were considered. The outcome was falls, defined as ‘falling on the ground or at some other level such as chair level’ over 12 months, recorded by researchers during face-to-face interviews. Of 855 women included in 12-month analysis, 39% had at least one fall over 12 months. The WHAS fall risk algorithm was derived by tree-based modelling and contained a short question on falls history, difficulties with balance during walking and with selected ADLs [21]. These items were used in the short PreFIT postal screener, with small modifications to make the response options across questions consistent and easy to complete in a postal format (Supplementary Files Table S1). Internal validation of the WHAS screener demonstrated equivalent or better performance than other well-known algorithms [5, 23].

### UK Prevention of Falls Injury Trial (PreFIT)

The PreFIT trial recruited 9,803 community-dwelling adults, aged at least 70 years, from 63 general practices across England. Trial protocol and results are published, including unadjusted prediction values [19, 20, 24]. We tested screening with onward referral to active falls prevention intervention (exercise or multifactorial assessment) compared to an advice leaflet only control, on outcomes of falls and fractures over 12 and 18 months. Trial participants were respondents to a mailed invitation sent to a random sample of older people registered in general practice and cohorts were assembled before randomisation, as per cluster trial design. All participants completed a detailed postal baseline health questionnaire prior to randomisation (*n* = 9,803), before one third of practices were randomised to control only (*n* = 3,223 participants; 33%) and two-thirds of practices (*n* = 6,580; 67%) allocated to postal screening with onward referral to treatment for those deemed at higher risk of falling. We report characteristics of the PreFIT and WHAS cohorts as per TRIPOD requirements (Supplementary Files Table S2).

### PreFIT baseline questionnaire (*n* = 9,803)

The baseline questionnaire collected demographic data, self-reported height, weight, falls, fractures, cognition, frailty, ADL difficulties, frequencies of problems with balance and health-related quality of life. Full details of definitions and standardised measurement scales used have been published [24]. In brief, cognition was assessed using a clock draw test [25], frailty was measured using the Strawbridge Frailty Index (SFI) [26] and quality of life measured using the Short-Form-12 version 2 (Physical/Mental Component Score; PCS/MCS) [27]. It was possible to reconstruct the postal screener using data items from the baseline questionnaire; these variables were used in the prediction models described below.

### PreFIT postal screener (*n* = 6,580)

The PreFIT postal screener was posted out by and returned to, general practices within a few weeks of return of the baseline questionnaire, to the subset of participants within practices randomised to active treatment (*n* = 6,580). The postal screener was short, being one side of A4 and size 12 Arial font. Draft versions were reviewed by patient representatives, general practitioners and by external monitoring committees. We predicted risk of future falling, over 12 months, based on screener responses, as lower risk (no previous fall, no balance difficulty and no/occasional ADL difficulty), or higher risk (any balance/ADL difficulty or one or more falls in the previous year (Supplementary Files Fig S1).

### Dependent variables

We classed people as having no falls, one fall or recurrent falls (≥ 2 or more) in the 12 months after practice randomisation. We defined a ‘fall’ using an internationally agreed definition, as per the Prevention of Falls Network Europe (PRoFaNE) [28] and used retrospective reporting in postal follow-up questionnaires at four, eight and 12 months, with each questionnaire having a recall period of four months. Fall reporting was compared to prospective reporting using monthly fall diaries over a four month period [29]. Fractures over 12 months were captured from NHS Digital Hospital Episode Statistics, including ICD-10 codes for all fracture diagnoses identified in hospital admissions, accident and emergency and clinic datasets [30]. We also searched general practice records for consultations for fractures and X-ray reports. Fracture events were classified according to PRoFaNE, plus rib, sternum, skull and facial fractures; all events were independently verified by study clinicians [19].

### Statistical analysis

We examined characteristics of respondents and non-respondents to the postal screener, using t-tests and chi-squared tests. As there were no differences in falls and fracture rates between trials arms at 12 months [20], treatment arms were collapsed. We used the control arm dataset (*n* = 3,223) to estimate the predictive performance of the screener items (falls, difficulties with balance and/or ADLs) recorded in the baseline questionnaire (Model 1). For Model 1, all baseline variables were entered and tested in univariate analyses. In Model 2, we estimated predictive performance of the postal screener in ‘practice’ in the intervention arm participants who returned the screener (*n* = 5,779/6,580). All variables were retained in subsequent models. In Model 3, we used the control group as a development data set to identify additional candidate predictors associated with falls and fractures over 12 months, and in Model 4 we used the intervention groups to undertake an internal validation (type IIa) of the expanded model [18].

We assessed model performance in all models using calibration and discrimination as per Collins et al. [18] and others [31]. Calibration is the agreement between observed and predicted probabilities of events and discrimination is the ability of a model to distinguish between participants who did and did not have an event over the study period [18, 31–33]. Those with the outcome event should have a higher predicted risk compared to those who do not. We assessed calibration using calibration plots, with observed risks plotted by quintile of predicted risks, and Cox calibration regression created by regressing the outcome on the predicted probability. The calibration plot reports the intercept and slope of the regression line, where zero intercept and slope of 1 indicates perfect calibration. Discrimination was assessed using the area under the receiver operating characteristic curve (ROC/AUC), whereby a value of 0.5 represents no better than chance and 1 represents perfect discrimination [31, 33]. We interpreted categorical AUC values according to recommended guidance: 0.90 to 1.00 as outstanding, 0.80 to < 0.90 = excellent, 0.70 to < 0.80 = acceptable/fair, 0.60 to < 0.70 = poor and 0.50 to < 0.60 as fail [34].

Logistic regression was used to identify additional candidate predictors. Predictors were analysed as continuous variables where possible to avoid loss of prognostic information. Remaining predictor variables were categorical, with SFI categorised as frail/non-frail [26]. Variables were excluded from regression models if they had high missingness (> 10%), evidence of high collinearity (*r* ≥ 0.8) with another predictor variable or had low event rates. However, item missingness was low (< 5%) in baseline questionnaires hence imputation methods were not used. There was a low proportion of missing data for outcomes of falls (5%) and fractures (< 0.01%). We did not test for interactions. We produced sensitivity–specificity plots for the development cohort, to visually display the optimal probability cut-offs, defined as the point where sensitivity and specificity curves cross. We also generated decision curve plots to calculate ‘net benefit’ in comparison to a default strategy of no treatment (screening) [35]. All analyses conducted on IBM SPSS version 23 and Stata SE 16. A two-sided *p* value < 0.05 was considered statistically significant.

## Results

Participant sociodemographic characteristics, quality of life, falls and fractures were well matched across the control and treatment groups (Table 1). We examined completion rates in those eligible to receive the postal screener (*n* = 6,580). Acceptability and item completion was high, with 88% returned to general practices (5,791/6,580), of which only 12 were returned blank (12/5,791; 0.2%). There were no differences in response to postal screener by age or sex, although non-responders were more likely to have a history of recurrent falls (166/801; 21%) compared to screener responders (920/5,779; 16%; *p* < 0.001), and had a slightly lower mean clock-draw test score (mean 5.4 (SD 0.9) versus 5.6 (SD 0.9); *p* < 0.001), and were more likely to be frail (212/801; 26%) versus 1,146/5,779; 20%) compared to those returning a screener (Further detail Supplementary materials Table S2).

**Table 1:** Characteristics of PreFIT model development and validation cohorts

		Control arm *N* = 3,223	Postal screener responders *N* = 5,779
Mean (SD), [missing]			
Age (years)		77.9 (5.7)	78.0 (5.7)
Body Mass Index (kg/m^2^)		26.4 (4.7) [104]	26.4 (4.5) [182]
Age left full-time education (years)		16.8 (4.6) [50]	16.8 (4.7) [83]
Clock Draw Test (0–6)		5.5 (0.9) [47]	5.6 (0.9) [105]
Age band, N (%)			
70–79		2,140 (66.4)	3,883 (67.2)
80–89		992 (30.8)	1,707 (29.5)
90–101		91 (2.8)	189 (3.3)
Gender, N (%)			
Male		1,557 (48.3)	2,728 (47.2)
Female		1,666 (51.7)	3,051 (52.8)
Living arrangement, N (%)			
Lives alone		1,048 (32.5)	1,906 (33.0)
Lives with others		2,155 (66.9)	3,842 (66.5)
Missing		20 (0.6)	31 (0.5)
GP Deprivation, N (%)			
Deprived (1–3)		603 (18.7)	1,125 (19.5)
Moderate (4–7)		1,566 (48.6)	2,490 (43.1)
Affluent (8–10)		1,054 (32.7)	2,164 (37.4)
Clock Draw test, N (%)			
0–3 (poor)		152 (4.7)	235 (4.1)
4–5 (moderate)		761 (23.6)	1,334 (23.1)
6 (excellent)		2,263 (70.2)	4,105 (71.0)
Missing		47 (1.5)	105 (1.8)
Difficulty with ADLs^**a**^, N (%), [missing]			
Taking a bath		464 (14.4) [155]	855 (14.8) [291]
Getting to the toilet		25 (0.8) [120]	31 (0.5) [156]
Dressing		51 (1.6) [107]	52 (0.9) [134]
Strawbridge Frailty Index^**b**^, N (%)			
Not frail		2,535 (78.6)	4,560 (78.9)
Frail		647 (20.1)	1,146 (19.8)
Missing		41 (1.3)	73 (1.3)
SF-12^**c**^, mean (SD), [missing]			
Physical component score		50.3 (10.2) [320]	50.5 (10.3) [540]
Mental component score		50.2 (9.3) [320]	50.5 (8.9) [540]
Balance difficulties, level surface, N (%)			
Never/Sometimes		2,923 (90.7)	5,288 (91.5)
Often/Very often/Always		280 (8.7)	467 (8.1)
Missing		20 (0.6)	24 (0.4)
Falls in last 12 months, N (%)			
No fall		2,179 (67.6)	3,917 (67.8)
Single fall		466 (14.5)	845 (14.6)
> 1 fall		500 (15.5)	920 (15.9)
Missing		78 (2.4)	97 (1.7)
Fall-related fracture in last 12 months, N (%)			
Yes		106 (3.3)	183 (3.2)
No		3,076 (95.4)	5,534 (95.8)
Missing		41 (1.3)	62 (1.0)
Risk of falling, baseline^**d**^ N (%)			
Low		1,839 (57.0)	3,,273 (56.6)
Intermediate		882 (27.4)	1581 (27.4)
High		500 (15.5)	920 (15.9)
Missing		2 (0.1)	5 (0.1)

No missing values unless where reported

^a^A lot of difficulty or unable to do

^b^SFI: not frail = none or problem in one domain only; frail = problem in 2 or more domains

^c^Short-Form 12 physical component score (PCS); mental component score (MCS)

^d^Risk of falling from baseline questionnaire

At 12 months, follow up rates to postal questionnaires were good, with 83% (8,136/9,803) returned. Of these, 41% (3,349/8,136) of participants had fallen by one year, 13% (1,094/8,136) were recurrent fallers and 3% (260/9,802) had sustained a fracture. We had complete fracture data except for one participant who withheld consent for access to routine health data (1/9,803). Total event rates were high, with 10,743 falls reported by 9,230 participants over 12 months. Over one third of participants, 3,349/9,230 (36%), had at least one fall. There were 1,808 multiple fallers reporting a total of 9,202 falls. Fracture event rate was also high, with 307 fractures amongst 260 participants over 12 months; of these, the most common sites were wrist/hand/forearm fractures (*n* = 77 participants: 81 fractures) and hip fractures (*n* = 60 participants: 61 fractures).

Overall, discrimination of Model 1 for the prediction of any fall over 12 months using the screener items in the questionnaire was AUC of 0.66 (CI 95% 0.64, 0.68; *p* < 0.001, poor), and for recurrent falls (AUC 0.70, 95% CI 0.68, 0.72; *p* < 0.001, fair) (Table 2). Prediction of fractures was poor (AUC: 0.60; 95% CI 0.54 to 0.65; *p* < 0.001). For the postal screener (Model 2), the discrimination was slightly better (Table 2).

**Table 2:** Prediction of falls and fractures over 12 months by development and validation datasets

	**N**	**AUC**	**95% CI**	***P* value**
**Any fall**				
Model 1, control, screener items only	2,661	0.66	0.64, 0.68	< 0.001
Model 2, intervention, postal screener only	4,841	0.67	0.65, 0.68	< 0.001
Model 3, control, development, full model*	2,329	0.70	0.68, 0.72	< 0.001
Model 4, intervention, full model**	4,240	0.71	0.69, 0.72	< 0.001
**Recurrent falls**				
Model 1, control, screener items only	2,574	0.70	0.68, 0.72	< 0.001
Model 2 intervention, postal screener only	4,712	0.71	0.69, 0.72	< 0.001
Model 3, control, development, full model	2,259	0.75	0.73, 0.78	< 0.001
Model 4, intervention, validation, full model	4,133	0.76	0.75, 0.78	< 0.001
**Any fracture**				
Model 1, control, screener items only	3,221	0.60	0.54, 0.65	< 0.001
Model 2, intervention, postal screener only	5,779	0.60	0.56, 0.64	< 0.001
Model 3, control, development, full model	3,078	0.73	0.67, 0.79	< 0.001
Model 4, intervention, validation, full model	5,503	0.71	0.67, 0.74	< 0.001

^*^Models 3 = development of expanded variable set, using control arm participant dataset, screener items from baseline questionnaire

^**^Models 4 = validation of expanded variable set using treatment arm dataset receiving postal screener

Additional predictors of falls identified in Model 3 included BMI, frailty (SFI), poorer physical and mental health (SF-12 PCS/MCS), (Table 3). The regression coefficients from the development dataset were then applied to the validation dataset, whereby discrimination improved (for falls AUC: 0.71; 95% CI 0.69, 0.72; *p* < 0.001, fair) (Table 2). Practice deprivation, female sex, frailty, poorer physical and mental health were predictive of recurrent falls (Table 3). Incorporating these variables also improved the discrimination for recurrent falls in the validation set (Model 4; Table 2) (AUC: 0.76; 95%CI 0.75, 0.78; *p* < 0.001). Prediction of fractures was only partially reliant on fall risk (Table 4), and was improved with the addition of age, sex and BMI (AUC 0.71; 95% CI 0.67, 0.74; *p* < 0.001; Table 2). Sensitivity–specificity plots for the development cohort for falls, recurrent falls and fractures over 12 months are displayed in the Supplementary Files (Figure S2 and FigureS3.)

**Table 3:** Logistic regression models for prediction of fall outcomes in control group (development) at 12 months

Predictors	Control group (model 1): Any fall *n* = 2329			Control group (model 1): Recurrent falls *n* = 2259		
	**Coefficient**	**95% CI**	***p*-value**	**Coefficient**	**95% CI**	***p*-value**
Age	-0.011	-0.029, 0.007	0.244	-0.002	-0.023, 0.019	0.831
Female gender	-0.019	-0.204, 0.167	0.844	-0.236	-0.464, -0.008	0.042
Lives alone	-0.034	-0.237, 0.169	0.745	-0.043	-0.288, 0.202	0.731
BMI	-0.020	-0.039, 0.001	0.044	-0.004	-0.026, 0.018	0.733
Practice deprivation 4 -7	0.099	-0.143, 0.340	0.424	0.367	0.064, 0.670	0.018
Practice deprivation 8 -10	-0.019	-0.275, 0.238	0.887	0.201	-0.119, 0.520	0.219
Clock Draw Test	-0.055	-0.171, 0.062	0.357	-0.099	-0.235, 0.038	0.156
SFI frail	0.333	0.080, 0.586	0.010	0.501	0.227, 0.774	< 0.001
SF-12 PCS	-0.019	-0.030, -0.009	< 0.001	-0.026	-0.039, -0.014	< 0.001
SF-12 MCS	-0.017	-0.027, -0.007	0.001	-0.027	-0.039, -0.016	< 0.001
High risk of falling	1.072	0.876, 1.267	< 0.001	1.293	1.053, 1.533	< 0.001
*Intercept*	2.592	0.521, 4.662	0.014	1.420	-1.004, 3.843	0.251

*Equation *^*1*^*:*$$logit\left[P\left(faller=1\right)\right]={\beta }_{0}+{\beta }_{1}age+{\beta }_{2} sex+{\beta }_{3}Living {+{\beta }_{4}BMI+\beta }_{5}Dep cat+{\beta }_{6}CDT+{\beta }_{7}SFI+{\beta }_{8}High risk+{\beta }_{9}PCS+{\beta }_{10}MCS$$

^1^For recurrent falls model, replace with $\left[P\left(recurrent faller=1\right)\right]$. Reference values: SFI frail vs. non-frail; GP deprivation score vs. 1–3; female vs. male; lives alone vs. lives with others; baseline high vs. low risk. The equation gives log odds. Probability can be calculated by taking the antilogit of the log odds

**Table 4:** Logistic regression models for fractures in control group (development) at 12 months

Predictors	Control group (model 1) 12 months, *n* = 3078		
	**Coefficient**	**95% CI**	**p**
Age	0.080	0.040, 0.121	< 0.001
Female gender	0.897	0.360, 1.434	< 0.001
BMI	-0.050	-0.105, 0.005	0.073
Practice deprivation 4 -7	-0.076	-0.784, 0.631	0.832
Practice deprivation 8 -10	0.344	-0.362, 1.051	0.339
Clock Draw Test	-0.022	-0.257, 0.212	0.851
High risk of falling	0.534	0.030, 1.038	0.038
*Intercept*	-9.641	-13.806, -5.477	< 0.001

*Equation:*$$logit\left[P\left(fracutre=1\right)\right]={\beta }_{0}+{\beta }_{1}age+{\beta }_{2} sex {+{\beta }_{3}BMI+\beta }_{4}Dep+{\beta }_{5}CDT+{\beta }_{6} High risk$$

Adjusted for these variables only due to predictor collinearity. Reference values: GP deprivation score vs. 1–3; female vs. male; baseline high vs. low risk. Example e.g. female, aged 73, BMI kg/m^2^ = 25, GP deprivation code 1–4, clock draw test 6 = fall risk is low. The equation gives log odds. Probability can be calculated by taking the antilogit of the log odds

Calibration plots were good, with slope values very close to 1, and models were well-fitted for prediction of falls, recurrent falls and fractures (see Supplementary Files Fig S3). These calibration plots show observed probability of falling (*y-*axis) over 12 months plotted against predicted probability of falling over 12 months (*x-*axis). Decision curve analyses suggested that the net benefit against threshold probability was high for falls and recurrent falls, less so for fractures.

## Discussion

We report the predictive utility of a short self-report postal screener suitable for administration to older people in the primary care setting. The postal fall risk screener was simple to administer but had limited accuracy in identifying those at higher risk of falling. According to accepted standards of interpretation, accuracy for single falls was poor, and repeat falls was acceptable/fair. We also examined whether addition of easy to collect sociodemographic and health-related variables could improve prediction. Additional variables improved all fall models to fair accuracy. This gives the possibility of extending current UK and international guidance for opportunistic screening for falls to a more population-based and systematic approach. The postal screener had poorer prediction of fracture risk. Fracture risk was only partially predicted by fall risk, but the addition of age, gender and BMI could improve performance of the short tool.

Our PreFIT postal screener incorporated risk factors previously identified within the USA WHAS [21]. We examined the characteristics of an ‘ideal’ predictive screening tool, by using recommended approaches to develop clinical prediction models statistically, using data from a derivation or development sample to re-test in a separate sample from a similar target populations [36] [32]. The WHAS was internally validated using appropriate statistical modelling but had yet to be tested in a large UK independent external dataset. We aimed to examine uptake and completion of a self-report postal screening version rather than a face-to-face, researcher or clinician-administered tool. The questions were short and understandable, asking about difficulties with balance whilst undertaking simple daily activities. Screener completion rates were high, with almost 90% returned to general practices.

Our short one-page screener had better prediction of those at greater risk of falling, people falling repeatedly over one year. This is perhaps unsurprising, but reassuringly, this is the group of fallers who are most likely to experience injury and decline in function [20]. Prediction improved slightly with adjustment for age, sex, frailty, physical and mental quality of life; lower mental health scores were predictive of falls. However, addition of all these items would expand the screener considerably, from three to 33 questions (SFI = 16 items, SF-12 = 12 items, age + sex = 2). This would undoubtedly impact upon completion and return rates, with minimal improvement in predictive utility. Prediction of fractures improved when adjusted for only age, sex and BMI (AUC 0.71), although this is still interpreted as ‘fair’ at best. We found evidence of collinearity between frailty and quality of life affecting fracture models, and these predictors were excluded. Few studies have tested the utility of predicting fall-related fractures, although several have examined injurious falls. Overall, studies are mixed, possibly due to low sample sizes, low event rates and variability in definitions for extent and type of injury [37].

A thorough, high-quality systematic review completed by Gade and colleagues identified 72 different prognostic models in 30 studies predicting falls in community-dwelling older adults (including the WHAS development cohort), although only three models had been externally validated, using data not used in original model development [38]. Overall risk of bias was deemed high, due to lack of standardised definitions for falls, low sample sizes and statistical concerns over model performance. Of the three validated models reported by Gade, their AUC values ranged from 0.62 to 0.69.

Our analytical approach adheres to TRIPOD recommendations for development and validation of prediction models [18]. Prediction models are widely used to augment rather than replace clinical decision making, to inform treatment or the need for further testing [32]. Risk tools either generate a continuous score used to estimate cumulative risk or yield a categorical score whereby risk status is classified as being at risk or not. We recommend recent explanatory guides for the application of prediction models in the clinical setting (e.g. [39]). Within falls prevention, these decisions are typically binary and thus require decision thresholds that are clinically relevant [33]. The optimal cut-offs for screeners should be chosen according to the relative costs of administration and subsequent referral for treatment, based on consideration of false positives and false negatives. In the context of falls, misclassification of those at lowest risk (false positive) may be less of an issue than the inaccurate underestimation of those at highest risk of falling (false negatives).

It is important for clinicians to understand the populations in whom models are developed and validated, bearing in mind any differences between source and test population characteristics. Item selection for our postal screener was informed by the WHAS dataset from another setting, undertaken in a female-only US sample of older women with mild disability. PreFIT included both sexes without restriction by upper age, functional or cognitive ability. Our large sample size of almost 10,000 older people from rural and urban settings across England, with an age range spanning thirty years, from 70 to 101 years, included older people with morbidity and disability. We used this comprehensive trial dataset to develop and validate our prognostic risk models using a split-sample validation approach (type IIa), as outlined by TRIPOD [18]. Calibration was excellent, although this may be expected given that a similar recruitment approach was used across groups in our sample.

We carefully considered our testing approach given demands to improve methodological quality and reporting standards of prognostic risk models. A systematic review of 120 prediction models found that the median number of predictors included in statistical models was six, median sample size 795 and median number of outcome events was 106 [32]. Our sample size was high and median number of events far exceeded those in other fall risk prediction studies (> 10,000 falls; > 300 fracture events) when compared to the findings of this systematic review. Many existing falls assessment tools were developed using expert clinical consensus or by selection of associative factors from cross-sectional studies rather than by formal quantitative testing of risk factors identified in prospective cohorts. Although considered modest (fair to poor), our ROC values are comparable with the US Elderly Accidents, Deaths and Injuries (STEADI) fall risk screener which reports AUC values of 0.64, although as with our findings, STEADI models also improved (to AUC 0.67) with the addition of sociodemographic characteristics [6]. The authors of STEADI argue that the sensitivity and specificity of their algorithm, interpreted as having moderate predictive validity, was tempered by the simplicity of measurement and adaptability for large scale survey purposes. Yet STEADI is a much longer screener tool and involves face-to-face assessment and balance tests. Extensive face-to-face objective testing is overly complex for routine use in primary care services on a population level. Muir et al. [40] argued that self-reported balance problems are on a par with more detailed measures and assessments of postural stability, such as observational gait and tandem stance tests.

Factors affecting sensitivity and specificity include prevalence and length of observation [41]. Unlike conventional screening for disease presence or absence, fall-related risk factors can change quickly over time and an important question for clinical recommendations is at what point should screening be repeated in older adults? Most hospital-based studies range from days to weeks of follow-up, compared to primary care or cohort studies that generally have longer time frames spanning a year or more. Longer-term monitoring may be required for those at higher risk and one option could be to undertake annual re-administration of the short screener within primary care, to identify the subgroup most likely to benefit from falls prevention services.

The PreFIT screener also compared well to other established clinical prediction tools in other areas of medicine. Higaonna [41] reported that, even in the acute hospital setting, no hospital-based fall screening tool exhibited both sensitivity and specificity of > 0.70, the minimum predictive validity criteria suggested by Oliver et al. [42]. Alba and colleagues [31] systematically reviewed the discriminatory capacity of prediction tools for: stroke risk in patients with atrial fibrillation (AUC range 0.66 to 0.75); risk of prostate cancer (AUC 0.56–0.74); mortality in people with heart failure (AUC 0.62–0.78), and adverse events in adults after discharge from emergency departments (AUC 0.58–0.64). These predictive tools were validated in at least five or more different external cohorts.

### Strengths

PreFIT had good geographical spread across England, with excellent representation of the ‘oldest old’ living independently, with a third being aged 80 years or older (*n* = 3,248). We used standardised definitions for outcomes as per international recommendations (e.g. PRoFaNE [28]), outcome assessors were blinded to all baseline predictors and data missingness was low (95% and 99.9% for falls and fractures, respectively). We interpreted AUC values cautiously, according to statistical guidance [34]. Current international guidance recommends at least 200 events for prediction modelling and our falls analyses far exceeded the minimum requirement. [18, 32] Guidelines highlight that it is optimal to refine existing models rather develop new risk models, hence split-sample validation using risk factors from the WHAS dataset. Few falls prediction tools incorporate ADL difficulties, and it was feasible to screen for ability to complete basic or instrumental ADLs. Future research could test telephone administration the screener and it is also feasible for use by those with limited clinical training.

### Limitations

Our analytical approach adheres to TRIPOD Type IIa, random split-sample development and validation [18]. The next phase of research would be to undertake full external validation (type 4) testing of the PreFIT screener in a separate cohort of older adults. Nevertheless, our trial dataset was large and used cluster sampling (GP practices), whereby participants were recruited and completed baseline measures prior to randomisation thus risk of contamination was low. There is a risk that trial interventions were confounded with the risk of falls or fracture, although our main analysis did not identify a significant treatment effect on fracture outcomes. Although the postal screener was short and completion rate high, we did not impute data for the 12% who failed to return a screener. Moreover, response rate may be lower in a sample who have not consented to participate in a research study. Our sample included predominantly white, cognitively intact community dwelling older adults, with a small proportion (< 5%) scoring lower on the clock drawing test (0–3). This may have impacted on the high response and completion rate. Representation of Black and minority ethnic groups within our recruited sample was lower than the English population [20]. Finally, we analysed falls events reported in postal questionnaires with four-month fall recall periods rather than falls reported in prospective monthly diaries, due to our observation that attrition increased over time when using falls diaries over 12-months [29]. We accept that recall bias and under-reporting of falls is a potential limitation.

## Conclusion

We found that a short postal screener can predict falls amongst community-dwelling older people at higher risk of falling, but only with fair accuracy for recurrent falls. Although falls prediction improved with addition of frailty, mental and physical health-related quality of life questionnaires, the gain was marginal against the cost of a longer screener. Prediction of fractures over 12 months improved when adjusted for age, sex and BMI. These variables are simple to collect in the community setting. There is potential for our short postal screener to be used in UK primary care to identify those most at need of referral for further specialist clinical and social support. Nevertheless, a simple screening tool for falls injury with better predictive values is still needed.

## Supplementary Information

**Additional file 1: Figure S1.** PreFIT Risk of falling algorithm. **Figure S2.** Sensitivity-specificity plots for prediction of falls, recurrent falls and fractures over 12 months for the development cohort. **Figure S3.** Calibration plots, observed vs expected probability of falls, recurrent falls and fractures over 12 months^1^. **Table S1.** PreFIT postal balance screener. **Table S2.** Comparison of original WHAS development cohort and PreFIT sample characteristics. **Table S3.** Comparison of characteristics of recruited vs analysed cohorts. **Figure S4.** Decision curve analyses plotting net benefit against threshold probability

## Data Availability

All requests for data should be sent to the corresponding author. Access to the available anonymised data may be granted following review.

## Abbreviations

ADL: Activities of daily living
AUC: Area under curve
BMI: Body mass index
FRAST: Falls risk assessment and screening tool
GP: General practitioner
ISRCTN: International standard randomised controlled trial number
PreFIT: Prevention of falls injury trial
PRoFaNE: Prevention of falls network Europe
ROC: Receiver operating characteristic
SF-12 PCS/MCS: Short-Form-12 Physical/Mental component score
SD: Standard deviation
SFI: Strawbridge frailty index
TRIPOD: Transparent reporting of a multivariate prediction model for individual prognosis or diagnosis
WHAS: Women’s health and ageing study
