# Supplementary material for: Falls and fracture risk screening in primary care: update and validation of a postal screening tool for community dwelling older adults recruited to UK Prevention of Falls Injury Trial (PreFIT)
Source: BMC Geriatr. 2023 Jan 23;23:42. doi: 10.1186/s12877-022-03649-5 (PMC9872287; doi:10.1186/s12877-022-03649-5)
Supplement: Supplementary file 1 — Additional file 1: Figure S1. PreFIT Risk of falling algorithm. Figure S2. Sensitivity-specificity plots for prediction of falls, recurrent falls and fractures over 12 months for the development cohort. Figure S3. Calibration plots, observed vs expected probability of falls, recurrent falls and fractures over 12 months1. Table S1. PreFIT postal balance screener. Table S2. Comparison of original WHAS development cohort and PreFIT sample characteristics. Table S3. Comparison of characteristics of recruited vs analysed cohorts. Figure S4. Decision curve analyses plotting net benefit against threshold probability [file 12877_2022_3649_MOESM1_ESM.pdf]

SUPPLEMENTARY FILES – For online

Figure S1. PreFIT Risk of falling algorithm

Figure S1.

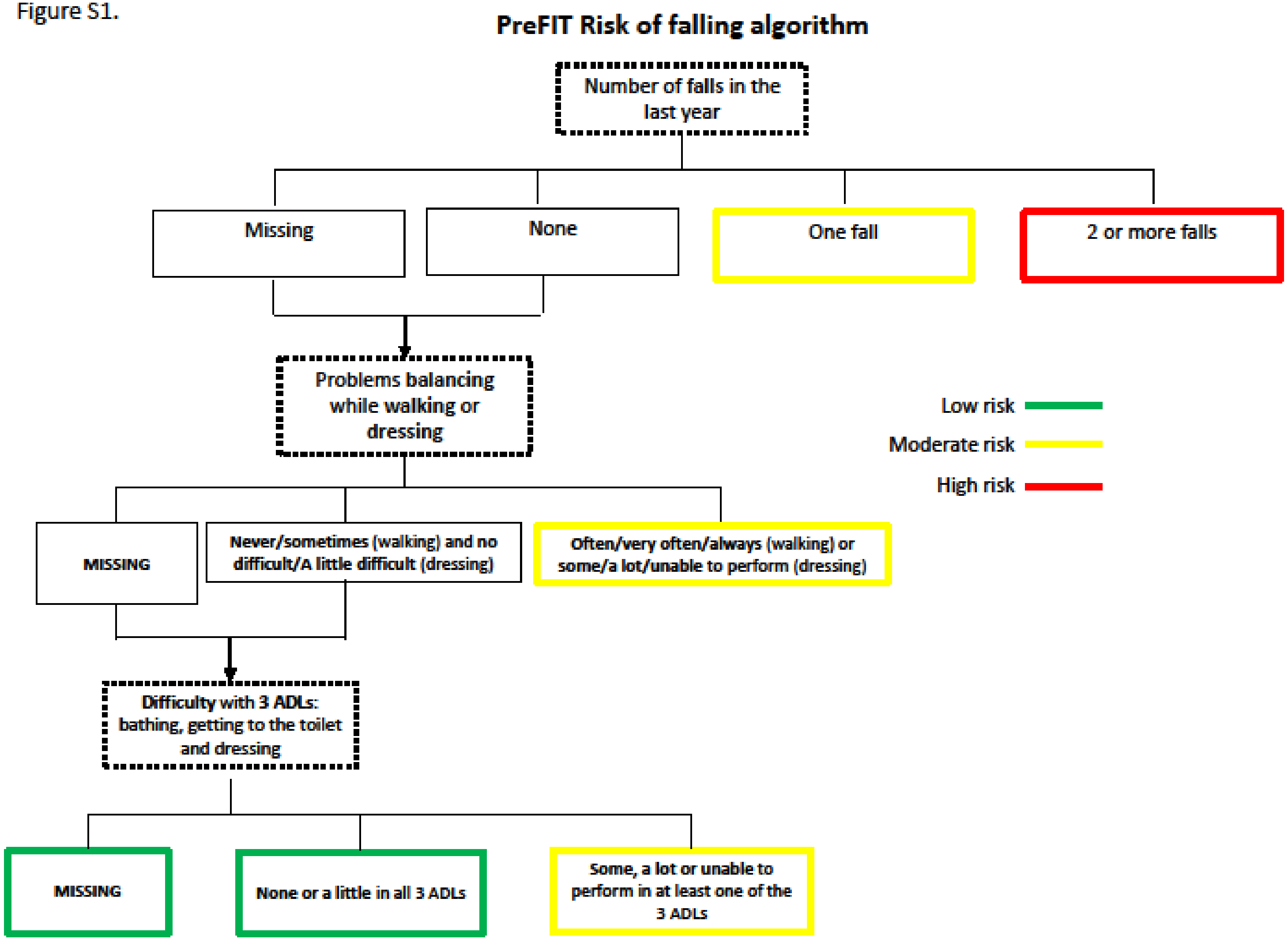

Figure S2. Sensitivity-specificity plots for prediction of falls, recurrent falls and fractures over 12 months for the development cohort.

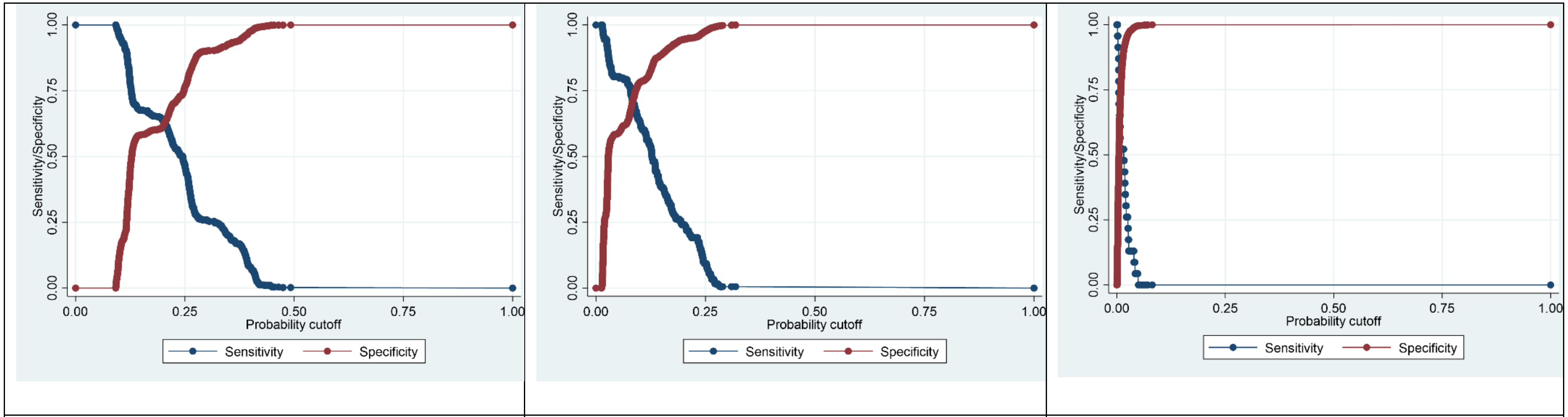

a) Falls
b) Recurrent falls
c) Fractures

Figure S3. Calibration plots, observed vs expected probability of falls, recurrent falls and fractures over 12 months[1]

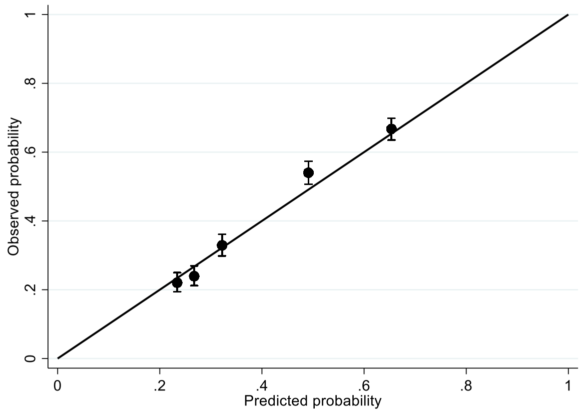

a) Falls (calibration-in-the-large is 0.08 and calibration slope is 1.13)

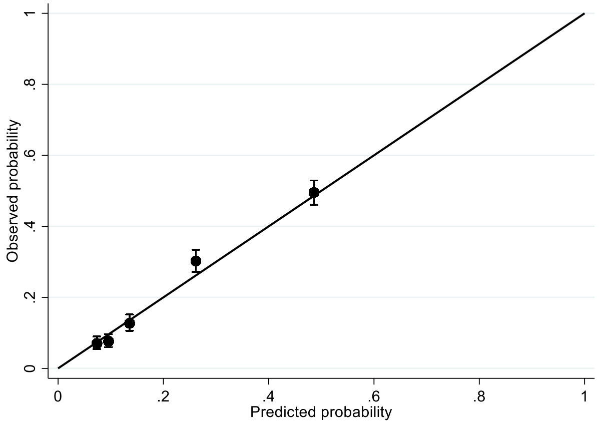

b) Recurrent falls (calibration-in-the-large is 0.08 and calibration slope is 1.05)

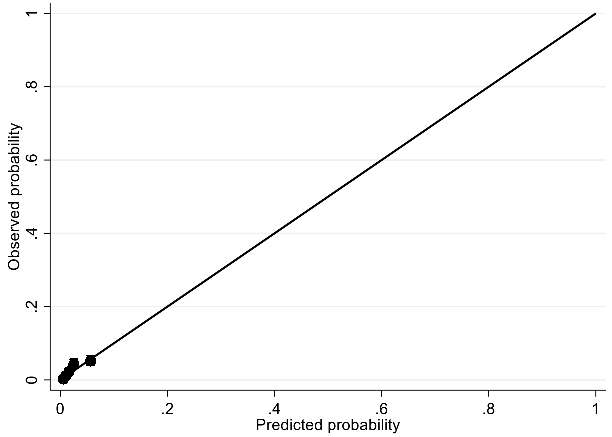

c) Fractures (calibration-in-the-large is -0.5 and calibration slope 0.81)

Table S1. PreFIT postal balance screener

Table S1. Postal screener

Your Balance and Mobility

We would be grateful if you could complete the questions below. Once you have completed the short survey, please return this form to the general practice using the stamped addressed envelope provided.

Q1. Please read the following statements and tick one

My balance is good and I want to keep it that way ☐

My balance is quite good but I would like to improve it ☐

I have some problems with balance that I want to overcome ☐

Q2. In the last 12 months, have you had any fall including a slip or trip following which you have come to rest on the ground, floor, or lower level? Please tick one box below that applies to you.

I have not fallen in the last year ☐

I have fallen once in the last year ☐

I have fallen more than once in the last year ☐

Q3. Do you have any difficulties with your balance whilst walking or dressing?

No, or just occasionally ☐

Yes, often or always ☐

Q4. Do you have any difficulties with daily activities, such as getting dressed, going to the toilet or taking a bath?

No, or just occasionally ☐

Yes, often or always ☐

If you do have problems with your balance, your GP may decide to refer you to another service for further advice and treatment.

In case you do need further assessment, please provide your telephone number.

Telephone Number:

Please return this form to your practice in the envelope provided.
Thank you for taking the time to complete this form.

PreFIT Study Group

@Copyright University of Warwick

Table S2. Comparison of original WHAS development cohort and PreFIT sample characteristics

Characteristic	WHAS original development cohort<br>N = 855	PreFIT trial cohort<br>N = 9803
Mean age (SD)	78 (8.1)	77.9 (5.7)
% Female	855 (100%)	5150 (52%)
Cognition	MMSE: Mean 26 (3.0)	CDT: Mean 5.5 (0.9)
Difficulty with ADLs	585 / 832 (70%)	-
Taking a bath	-	1466 (15.0)
Getting to the toilet	-	67 (0.7)
Dressing	-	126 (1.3)
Any falls in previous year	279 / 830 (34%)	3150 / 9803 (32%)
Mean BMI (SD)	29 (6.9)	26.5 (4.6)

Difficulty with ADLs =A lot of difficulty or unable to do; MMSE= mini-mental state examination, score out of 30; Clock Drawing Test, score out of 6.

Table S3. Comparison of characteristics of recruited vs analysed cohorts

	Control (development) group		Treatment (validation) group	
	**All recruited n = 3223**	**Analysed sample n = 2662 (83%)**	**Screener responders n = 5779**	**Analysed sample n = 4841 (84%)**
	**Mean (SD) [missing]**	**Mean (SD) [missing]**	**Mean (SD) [missing]**	**Mean (SD) [missing]**
Age (years)	77.9 (5.7)	77.6 (5.5)	78.0 (5.7)	77.8 (5.6)
Body Mass Index (kg/m^2)	26.5 (4.7) [104]	26.4 (4.8) [67]	26.4 (4.5) [182]	26.4 (4.5) [129]
Age left FT Education (years)	16.9 (5.2) [50]	16.8 (4.6) [28]	16.9 (5.0) [83]	16.8 (4.5) [62]
Clock Draw Test (0-6)	5.5 (0.9) [47]	5.6 (0.9) [34]	5.6 (0.9) [105]	5.6 (0.8) [74]
**Age band, N (%)**				
70-79	2140 (66.4)	1831 (67.7)	3883 (67.2)	3317 (68.5)
80-89	992 (30.8)	800 (30.1)	1707 (29.6)	1382 (28.6)
90-101	91 (2.8)	61 (2.2)	189 (3.3)	142 (2.9)
**Gender, N (%)** Male	1557 (48.3)	1276 (47.9)	2728 (47.2)	2320 (47.9)
Female	1666 (51.7)	1386 (52.1)	3051 (52.8)	2521 (52.1)
**Living arrangements, N (%)**				
Live alone	1048 (32.5)	859 (32.3)	1906 (33.0)	1555 (32.1)
Live with others	2155 (66.9)	1790 (67.2)	3842 (66.5)	3262 (67.4)
Missing	20 (0.6)	13 (0.5)	31 (0.5)	24 (0.5)
**GP Deprivation, N (%)**				
Deprived (1-3)	603 (18.7)	496 (18.3)	1125 (19.5)	942 (19.5)

Moderate (4-7)	1566 (48.6)	1286 (48.3)	2490 (43.1)	2096 (43.3)
Affluent (8-10)	1054 (32.7)	880 (33.1)	2164 (37.5)	1803 (37.2)
**Clock Draw test**				
0-3 (poor)	152 (4.7)	109 (4.1)	235 (4.1)	162 (3.4)
4-5 (moderate)	761 (23.6)	610 (22.9)	1334 (23.1)	1100 (22.7)
6 (excellent)	2263 (70.2)	1909 (71.7)	4105 (71.0)	3505 (72.4)
Missing	47 (1.5)	34 (1.3)	105 (1.8)	74 (1.5)
**Difficulty with ADLs**[a]				
Taking a bath	464 (14.4)	359 (13.5)	855 (14.8)	674 (13.9)
Getting to the toilet	25 (0.8)	16 (0.6)	31 (0.5)	20 (0.4)
Dressing	51 (1.6)	34 (1.3)	52 (0.9)	37 (0.8)
**Strawbridge frailty**[b]				
Not frail	2535 (78.6)	2131 (80.1)	4560 (78.9)	3888 (80.3)
Frail	647 (20.1)	504 (18.9)	1146 (19.8)	896 (18.5)
Missing	41 (1.3)	27 (1.0)	73 (1.3)	57 (1.2)
**Balance difficulty on level surface**				
Never/Sometimes	2923 (90.7)	2444 (91.8)	5288 (91.5)	4470 (92.3)
Often/Very often/Always	280 (8.7)	205 (7.7)	467 (8.1)	354 (7.3)
Missing	20 (6.2)	13 (0.5)	24 (0.4)	17 (0.4)
**Falls in last 12 months**				
No fall	2179 (67.6)	1804 (67.8)	3917 (67.8)	3280 (67.8)
Single fall	466 (14.5)	396 (14.9)	845 (14.6)	726 (15.0)

>1 fall	500 (15.5)	411 (15.4)	920 (15.9)	757 (15.6)
Missing	78 (2.4)	51 (1.9)	97 (1.7)	78 (1.6)
**Fracture in last 12 months**				
Yes	106 (3.3)	81 (3.0)	183 (3.2)	143 (3.0)
No	3076 (95.4)	2552 (95.9)	5534(95.8)	4650 (96.0)
Missing	41 (1.3)	29 (1.1)	62 (1.0)	48 (1.0)
**Risk of falling, baseline CRF[c]**				
Low	1839 (57.1)	1536 (57.7)	3273 (56.6)	2766 (57.1)
Intermediate-High	1382 (42.9)	1125 (42.3)	2501 (43.3)	2070 (42.8)
Missing	2 (0.1)	1 (0.04)	5 (0.1)	5 (0.1)

FT: Full Time education; [a]A lot of difficulty or unable to do; [b]Strawbridge not frail = none or problem in one domain only; frail = problem in 2 or more domains. [c]Risk from baseline CRF. Analysed sample comprises those who returned baseline and 12-month postal questionnaires.

Figure S4: Decision curve analyses plotting net benefit against threshold probability

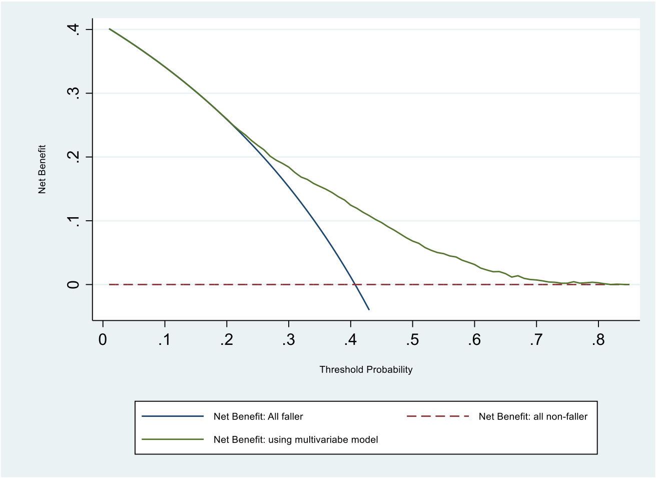

a) Falls

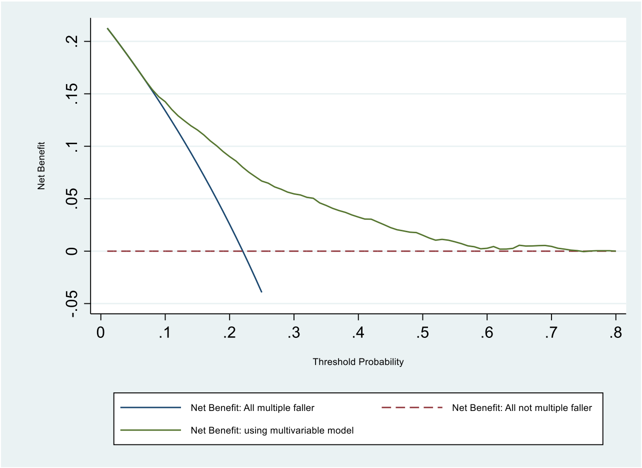

b) Recurrent falls

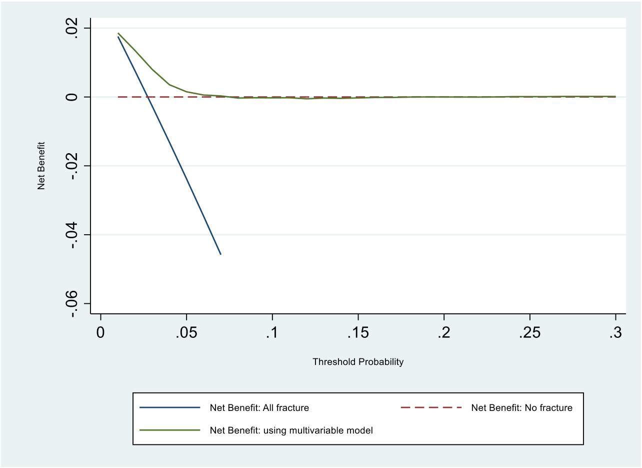

c) Fractures
